# Research progress of dengue virus T cell epitope mediated cellular immunity in avoiding ADE

**DOI:** 10.3389/fimmu.2026.1785887

**Published:** 2026-04-01

**Authors:** Mengye Ma, Peipei Ye, Jieting Pan, Yu Zhang, Weilei Yan, Bingqing Xu, Jiaxin Ding, Liming Jiang

**Affiliations:** 1 Health Science Center, Ningbo University, Ningbo, Zhejiang, China; 2 School of Basic Medical Sciences, Health Science Center, Ningbo University, Ningbo, Zhejiang, China; 3 Department of Hematology, The Affiliated People’s Hospital of Ningbo University, Ningbo, Zhejiang, China; 4 Institute of Hematology, Ningbo University, Ningbo, Zhejiang, China; 5 Yunnan Provincial Key Laboratory of Public Health and Biosafety, Yunnan Center for Disease Control and Prevention (Yunnan Provincial Academy of Preventive Medical Sciences), Kunming, China; 6 Health Laboratories Center, Yunnan Center for Disease Control and Prevention (Yunnan Provincial Academy of Preventive Medical Sciences), Kunming, China

**Keywords:** ADE, cellular immunity, dengue virus, T cell epitope, vaccine

## Abstract

Dengue virus (DENV), as a widely circulating arbovirus, is prone to causing dengue fever and poses a serious threat to human health. Nevertheless, there is currently no ideal safe and effective vaccine for DENV. In particular, vaccination with approved DENV vaccines may increase the chance of infection with heterotypic serotypes of DENV and the risk of severe dengue upon infection. Antibody-dependent enhancement (ADE) is considered a major mechanism contributing to severe disease in secondary infections, which seriously restricts the safety and efficacy of the vaccine. Notably, many studies have shown that DENV T cell epitopes induce cellular immunity by producing large amounts of cytokines, which may contribute to controlling DENV infection and potentially modulating ADE risk. However, the relationship between T cell responses and ADE is complex and requires careful balance to avoid immunopathology. Additionally, the novel mRNA-LNP vaccine has shown promise in preclinical models due to its superior stability and controllability. This review highlights the potential of T cell epitope-based vaccines to complement existing strategies of DENV vaccine development and provide new ideas for the prevention and treatment of DENV.

## 1 Introduction

Dengue virus (DENV) is an arbovirus widely distributed in tropical and subtropical regions, which is transmitted by Aedes aegypti and Aedes albopictus. It belongs to the Flaviviridae family and can be divided into four serotypes (DENV I-IV). The variation in amino acid sequences for different DENV serotypes is 30-35% (1). In addition, the virus contains a single-stranded positive-stranded RNA genome encoding three structural proteins [pre-membrane protein (prM), envelope protein(E), capsid protein(C)] and seven non-structural proteins (NS1, NS2A, NS2B, NS3, NS4A, NS4B, and NS5). DENV spreads in more than 100 countries and causes approximately 390 million cases of infection each year, with an upward trend in recent years (2). Generally, DENV infection easily leads to dengue fever and has extensive clinical manifestations. According to the 2009 WHO guidelines, dengue fever was classified into non-warning symptoms, warning symptoms (persistent vomiting, abdominal pain, low platelet count, and clinical effusion), and severe dengue fever (severe plasma leakage, bleeding, and organ involvement) (3).

**Figure 1:**
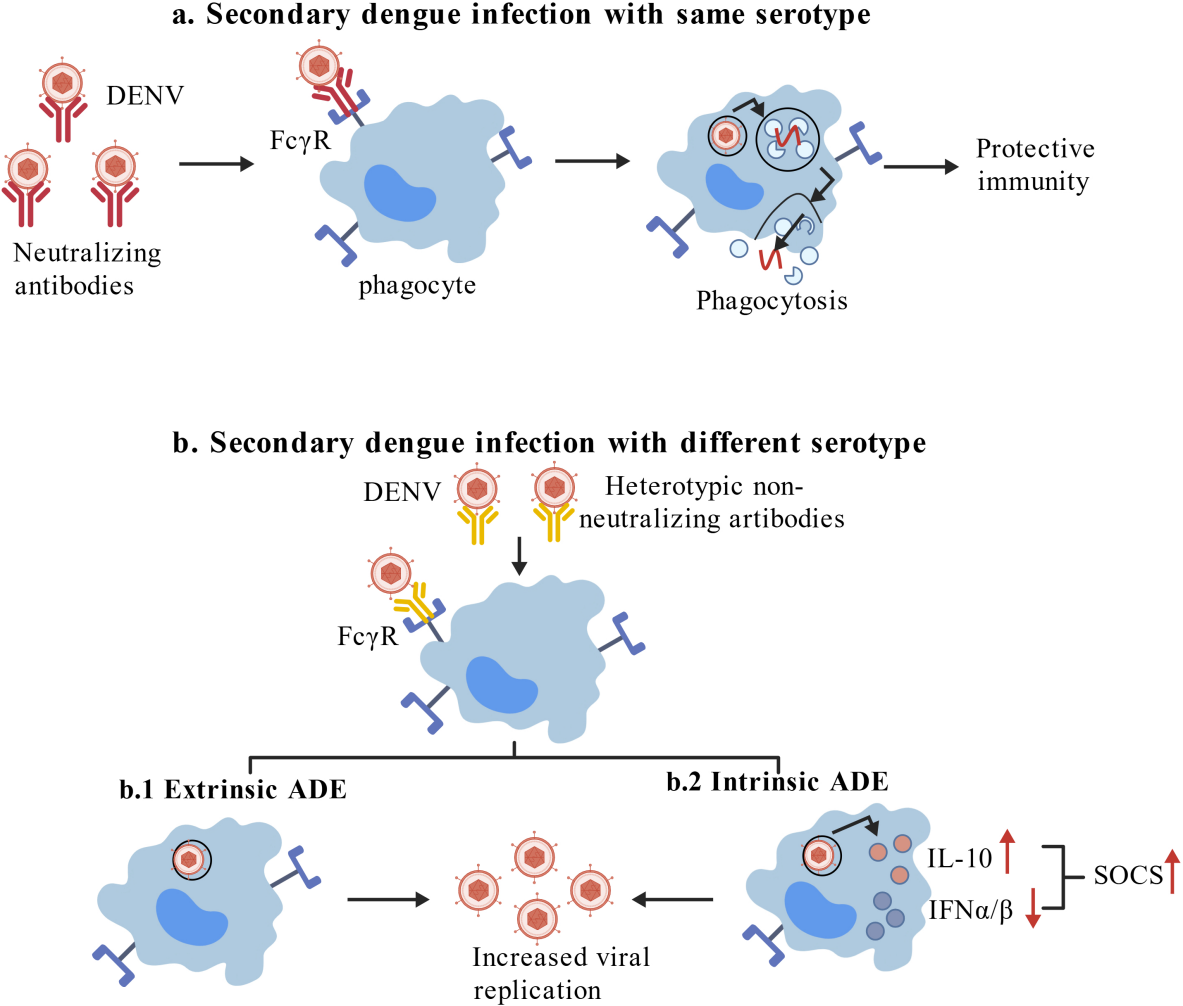
**(a)** Secondary dengue infection with the same serotype; **(b)** secondary dengue infection with different serotype; b.1 mechanism of action of extrinsic ADE; b.2 mechanism of action of intrinsic ADE [modified from Rahul Shukla (5)].

Generally, the occurrence of severe dengue fever is related to many factors. Predominantly, it’s particularly closely related to antibody-dependent enhancement (ADE). ADE is a special phenomenon whereby patients have different serotypes of DENV in primary and secondary infections. This means that the primary infection antibodies are at a sub-neutralizing level in the host, which then promotes virus replication during secondary different DENV serotypes. Sub-neutralizing heterotype DENV antibodies promote the entry of DENV into monocytes and macrophages and facilitate DENV entry into cells via the Fcγ receptors, which improve the uptake ability of host cells for DENV and promote virus replication. For example, DHF/DSS is at the highest risk following secondary DENV-2 infection after primary DENV-1 infection, while the pathogenicity of the DENV-2/DENV-3 infection sequence is lower. This differential risk is closely related to ADE. After initial infection with DENV-1, cross-reactive antibodies fail to effectively neutralize heterologous DENV-2, but instead form infectious immune complexes that enter Fcγ receptors, leading to increased viral replication and elevated viremia. This expanded pool of infected cells subsequently stimulates T cell-mediated immunopathological effects. In contrast, the DENV-2/DENV-3 infection sequence does not induce cross-reactive antibodies with potent ADE activity, thus exhibiting lower pathogenicity (4). Similarly, DENV bind to antibodies through Fcγ receptors and downregulate DENV specific pattern recognition receptor (PRR) signaling, which inhibit the release of type I interferon (IFNα/β) and activate the production of interleukin-10 (IL-10), thus leading to the upregulation of the suppressor of cytokine signaling (SOCS) family to creating a favorable environment for DENV replication and increasing the host viral load, that is intrinsic ADE (5) (**Figure 1**). For traditional DENV vaccines, the induced neutralizing antibodies didn’t effectively neutralize the heterotype DENV, instead promoting infection and worsening of the condition, which increases the difficulty of vaccine development and vaccination risks. Therefore, ADE is an important bottleneck restricting the development of DENV vaccines.

**Figure 2:**
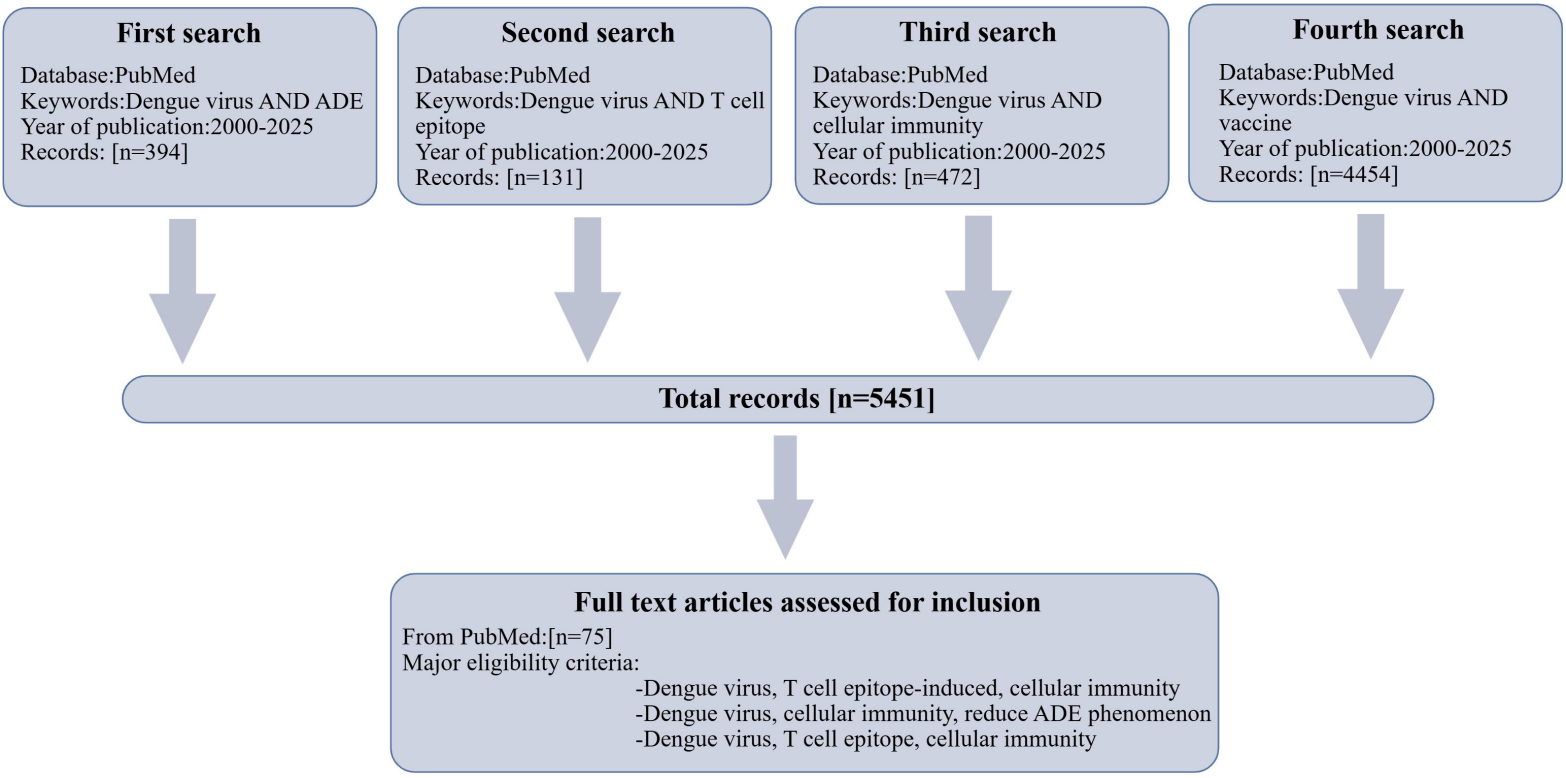
Literature search and inclusion methods: We use the PubMed search engine to identify dengue virus, T cell epitope, and ADE. PubMed search terms “Dengue virus and ADE”, “dengue virus and t cell epitope”, “ dengue virus and cellular immunity”, “dengue virus and vaccine” to search for articles published between July 15, 2000 and November 10, 2025. Finally, 75 articles addressed at least one core theme and were included in the final review.

Compared with the ADE problem caused by humoral immunity, moderate cellular immunity may lead to different results. Numerous studies have confirmed that DENV T cell epitope-induced T cell immunity is associated with reduced ADE between DENV and flavivirus (6, 7). T cell immunity can contribute to viral clearance through cytotoxic effects or cytokine secretion to weaken the ADE phenomenon by clearing infected cells in the presence of enhancing antibodies (8). Zellweger et al. reported that DENV-specific CD8^+^ T cells induced by DENV infection can prevent ADE and dramatically reduce viral load in mouse models (8). Preclinical studies have shown that the mRNA LNP vaccine encoding DENV prM-E or E-DIII/NS1 proteins can induce serotype-specific neutralizing antibody and T cell response in a mouse model, protect against homologous virus challenge, and attenuate ADE responses compared to immune sera from wild-type infection (9, 10). In addition, T cell responses are not uniformly protective. Excessive or dysregulated T cell activation can trigger cytokine storms, which are characterized by elevated levels of IFN - γ and TNF - α. These factors are considered to be important causes of severe dengue hemorrhagic fever. At the same time, the original antigenic sin(OAS) at the T cell level may lead to the deviation of immune memory orientation, resulting in T cell targeting errors and damage to virus control (11). These findings remain to be validated in human clinical trials, as direct evidence that T cell-epitope vaccines reduce ADE events in humans is currently lacking. Consequently, the induction of broad-spectrum neutralizing antibodies against all four serotypes still needs a multivalent vaccine design, such as the E-DIII and NS1 chimeric strategy, and the heterologous protective effect and long-term immune persistence need to be further verified. In order to systematically elaborate, T cell epitopes induce cellular immunity to reduce ADE in heterotype DENV infection. This review aims to summarize whether the cellular immunity induced by dengue virus T cell epitopes can prevent and reduce the ADE phenomenon, and explore the prospects of broad-spectrum mRNA-LNP vaccines based on conserved T cell epitopes. As a narrative review, we selectively cited literature from approximately 2000 to 2025 that provides mechanistic insights into DENV immunology, T cell epitope characterization, and vaccine development (**Figure 2**). Key references were identified through PubMed searches using terms such as “Dengue virus and ADE”, “Dengue virus and T cell epitopes”, “Dengue virus and cellular immunity”, and “Dengue virus and vaccines”, supplemented by seminal papers in the field identified through citation tracking and expert knowledge.

## 2 The “joys” and “worries” of dengue vaccine research

DENV is a major threat to global public health, and there is currently no specific available treatment. Among them, the ADE phenomenon, primarily driven by sub-neutralizing antibodies, and the potential for T cell-mediated immunopathology in some contexts, present challenges to vaccine safety and efficacy (12). Although there is no ideal dengue vaccine widely recognized, some progress has been made in the following candidate vaccines: live attenuated vaccines, inactivated vaccines, recombinant subunit vaccines, DNA vaccines, mRNA vaccines, monoclonal antibody vaccines, adenovirus vector vaccines, multivalent synthetic peptide vaccines, and antiviral small-molecule drugs.

### 2.1 Live attenuated vaccine

Live attenuated vaccines have non-pathogenic and immunogenic characteristics, inducing the body to produce specific B-cell and T-cell responses with strong immunity, which can enable the body to obtain long-term or lifelong protection. It has been considered a promising scheme for the development of a dengue vaccine. However, with the progress of research, relevant studies have shown that certain live attenuated vaccines lack T cell immune response against the non-structural antigen of DENV, which was associated with the immune efficacy and increased ADE risk in specific populations (13). For example, the chimeric tetravalent dengue vaccine CYD-TDV, developed by Sanofi Pasteur, as the world’s first clinically approved dengue vaccine, was approved for marketing in some Latin American and Asian countries (14). Nevertheless, the results of the long-term safety retrospective analysis showed that the vaccine had low efficacy (30.2-60.8% overall, 44.6% in children ≤ 9 years vs. 65.6% in >9 years)for children ≤ 9 years old, and increased hospitalization risk (hazard ratio 1.58, 95% CI 0.83-3.0) in seronegative patients, which posed great potential safety risks (15). Consequently, the vaccine is only used for patients aged 9–45 years old with seropositive status (13).

Unlike CYD-TDV, which primarily induces structural protein-targeted B cell immunity and limited NS-specific T cell responses, the Takeda TAK-003 vaccine can induce both humoral and cellular responses with persistent antibodies and multifunctional T cell responses, including CD8^+^ and CD4^+^ T cells targeting both structural and non-structural proteins, which may contribute to reduced ADE risk compared to CYD-TDV, though clinical validation remains ongoing (16). Similarly, TV003/TV005, which is currently undergoing phase 3 clinical trials in Thailand, Brazil, and other places, contains complete nonstructural proteins of three of the four DENV serotypes (DENV-1, -3, 4) and has been shown to induce multifunctional CD8^+^ T cell responses directed against highly conserved epitopes (17). Among them, TV003 can achieve 74% quadrivalent serum conversion (PRNT60 ≥ 1:10) with a single dose, and TV005 has been optimized to increase to 90%, both of which can induce sterile immunity defined by the absence of detectable viremia and no significant antibody titer boost (>4-fold) after a second vaccine dose. However, it is worth noting that this vaccine has limitations such as limited applicability to the population (disabled for immunocompromised individuals), incomplete coverage of DENV-2 non-structural proteins, missing quantitative data on heterologous cross neutralizing antibodies, and incomplete confirmation of severe protective efficacy at the Phase 3 population level (17). Moreover, these vaccines demonstrate that current live attenuated platforms can elicit T cell immunity, yet they still exhibit limitations. Some vaccines have imbalanced immunity to the four DENV serotypes, with significant differences in the induced antibody titers across serotypes, and the precise mechanisms of T cell responses to clinical outcomes remain to be fully established (18–20).

Moreover, these vaccines demonstrate that current live attenuated platforms can elicit T cell immunity, yet they still exhibit limitations. Some vaccines have imbalanced immunity to the four DENV serotypes, with significant differences in the induced antibody titers across serotypes, and the precise mechanisms and quantitative contribution of T cell responses to clinical outcomes remain to be fully established.

### 2.2 Inactivated vaccine

Compared with live attenuated vaccines, inactivated vaccines have higher safety. Nevertheless, during the inactivation process, the neutralizing epitopes of the virus may be destroyed, resulting in poor induction of neutralizing antibody responses. Generally, the immune efficacy is weaker than that of live attenuated vaccines, and the production cost is higher. Despite these limitations, inactivated vaccines remain a viable option for dengue vaccine development. For example, the quadrivalent purified inactivated candidate vaccine DPIV, which is being jointly developed by WRAIR, GSK, and Fiocruz, has good tolerability. A phase I clinical trial in the United States showed that 12 months after vaccination (M13), the seropositivity rates of neutralizing antibodies induced by different DPIV formulations against DENV-1 to -3 range from 11.8% (1μg+alum group for DENV-3) to 83.3% (1μg+AS03B group for DENV-1) (neutralizing antibody titer ≥10 detected by MN50 assay). As an inactivated vaccine, the immunogenicity of the four serotypes of DENV is relatively balanced, although cross-reactivity may affect vaccine efficacy, which requires further investigation (21). Similarly, TPIV has high immunogenicity and induces T cell and B cell immune responses. More notably, the neutralizing antibody titer can last up to 9 months after two doses of the vaccine. However, the neutralizing antibody titer rapidly decreases after vaccination in adult trials. In addition, the induced T cell response is relatively weak, potentially raising concerns about ADE risk (22).

### 2.3 Recombinant subunit vaccine

Recombinant subunit vaccines were made from specific antigens of the pathogen, which contain no whole pathogens and are non-infectious and highly safe. However, recombinant subunit vaccines cannot infect or replicate in host cells, and their inherent immunogenicity is limited. With the development of new adjuvants and vaccine delivery systems, vaccine efficacy, safety, and cost have all been improved, becoming a potential approach for the development of dengue vaccines. For example, as a recombinant subunit vaccine, the memory B cell immune response induced by V180 is relatively balanced among different serotypes of DENV (23). However, due to the limited T cell epitopes contained in the V180 vaccine, the cellular immune response is insufficient, which may weaken the vaccine’s protective efficacy and potentially increase the risk of severe disease in secondary DENV infections. Therefore, V180 as an independent vaccine may not be a feasible solution for preventing or treating dengue fever. Nevertheless, the development of formulations with ISCOMATRIX ™ adjuvants has improved the immunogenicity of V180 in Phase I clinical trials, enhancing antibody titers and meeting the prescribed positive immune response criteria, making it a potential candidate for further evaluation in the prevention and treatment of DENV (24).

### 2.4 DNA vaccine

The clinical application of DNA vaccines mainly faces the problem of poor immunogenicity. Currently, improvements are mainly made by adding adjuvants and replacing drug delivery systems, which are expected to become the development choice for new DENV vaccines. For example, TVDV is a DNA vaccine developed by the Naval Medical Research Center. The results of the Phase 1 population trial showed that although the vaccine produced a good anti-dengue T cell immune response, it had not yet reached optimal immunogenicity, and the antibody neutralization response was also poor (25). Porter et al. demonstrated that Vaxfectin^®^-adjuvanted tetravalent dengue DNA vaccine significantly enhanced neutralizing antibodies against DENV-1, -3, and -4 (2- to 10-fold) in nonhuman primates, but did not significantly improve IFN-γ ELISPOT-detected T cell responses. Vaccinated animals showed partial protection against DENV-2 challenge. Notably, while neutralizing antibodies are generally considered protective, sub-neutralizing antibody titers may theoretically increase ADE risk, and uncontrolled T cell activation can contribute to cytokine storm in severe dengue. The balance between protective immunity and potential immunopathology requires careful evaluation in clinical trials (26). In addition to the structural protein EDIII, DDV also targets the non-structural protein NS1, thus inducing a wide range of T cell responses in mice. While this strategy may theoretically enhance cross-serotype coverage, its ability to reduce secondary infection severity and avoid T cell-mediated immunopathology requires validation in clinical trials. D1ME100 expresses prM and E genes using the VR1012 plasmid vector (27). After Phase 1 clinical trials, the vaccine induced modest neutralizing antibody responses only at the highest dose (5mg) in a minority of subjects (<50%). As a monovalent DENV-1 vaccine, D1ME100 offers no protection against other serotypes. D1ME100 may have regional limitations, widespread cross-reactions, and theoretical concerns regarding secondary infection reactions in subjects with robust immune responses that harm the health of the vaccinated individuals.

### 2.5 mRNA-modified vaccine

The modified mRNA-LNP vaccine is encapsulated by lipid nanoparticles, targeting the prM and E protein genes encoding DENV, which can protect the vaccine from various enzymes in the host’s body and potentially enhance its long-lasting efficacy. In preclinical models, mRNA-LNP has shown that mRNA-LNP can provide a strong neutralizing antibody response and specific T cells against dengue fever in AG129 mice. It induces serotype-specific immunity at low levels of ADE, which helps AG129 mice with low immune function resist fatal DENV attacks (28). Compared with DNA vaccines (DENV1 neutralizing EC50≈1/100), attenuated live vaccines CYD-TDV (DENV1 neutralizing EC50≈1/60 in serum-negative individuals in phase III clinical trials) and TAK-003 (DENV1 neutralizing EC50≈1/184), this vaccine induced a DENV1 neutralizing EC50 of 1/420 at a dose of 10 μg, achieving effective cellular immune activation while inducing humoral immunity. However, the aforementioned traditional vaccines did not achieve such a balanced dual immune response. Notably, these findings are limited to animal studies, and human data on ADE reduction remain to be established. Thereby, mRNA-modified vaccines may offer hope for addressing the ADE phenomenon associated with DENV.

### 2.6 Virus vector vaccine

In the past few years, new pathogens have emerged and posed a serious threat to global health. As an emerging platform, viral vector vaccines induce strong humoral by delivering immunogens through recombinant viruses, which will become an important means of DENV prevention in the future. For example, CADVax-DenTV integrated the prM and E protein genes of DENV types I, II, III, and IV (29). Experiments in rhesus macaques demonstrated that the vaccine induces high-titer antibodies capable of neutralizing all four dengue virus serotypes *in vitro*. It blocked viremia by serotypes I and III, whilst providing weaker and delayed protection against serotypes II and IV (29). Besides, the immune effect can be enhanced by vaccinating with a second dose of booster vaccine or using different vaccines on the same platform. While viral vector vaccines demonstrate promising immunogenicity, mRNA-LNP vaccines offer distinct advantages in inducing robust CD4^+^ and CD8^+^ T cell responses due to their inherent adjuvant properties and efficient intracellular antigen presentation (30).

### 2.7 Multivalent synthetic peptide vaccine

Peptide vaccines are taken up by antigen-presenting cells and presented on the cell surface to induce cellular and humoral immunity. Although only a few peptide vaccines have been licensed so far, there is still great potential for the development of DENV vaccines. A selected HLA class I dengue peptide, synthesized by an N-terminal proteasome cleavage sequence (AAY) and a 1-mercaptopropionyl group, promotes its binding to Gold Nanoparticle (GNP). After purification, the PepGNP vaccine can be obtained (31). Notably, in healthy adults, peptide vaccines induce detectable CD $8^{+}$T cell immunity without inducing the production of related antibodies or strong humoral responses against the four serotypes of DENV in Switzerland. This approach may potentially reduce ADE risk, but the lack of neutralizing antibodies could limit sterilizing immunity. In addition, the response of CD8 ^+^ T cells in the high-dose group was poor. Moreover, the sample size of the experiment was small, only covering specific populations, and the long-term protective effect is unknown (31).

### 2.8 Others

Monoclonal antibodies and small-molecule antiviral drugs have emerged as promising avenues of research for the prevention and treatment of DENV infection. JNJ-1802 and JNJ-A07, developed by Johnson & Johnson Pharmaceutical Group, represent notable examples of anti-DENV small-molecule drugs. These drugs impede DENV replication by disrupting the interaction between the NS2B/NS3 protease/helicase complex and the NS4A-2K-NS4B cleavage intermediate, thereby blocking the *de novo* formation of vesicle packets (VPs), the sites of DENV RNA replication (32). JNJ-1802 has demonstrated efficacy in non-human primates, while JNJ-A07 is currently in the preclinical stage. *In vitro* studies indicate that these drugs have antiviral effects on all serotypes of DENV and also exhibit good metabolic stability in human and mouse liver microsomes. Further clinical development is warranted to fully evaluate their therapeutic potential. VIS513, a monoclonal antibody, is distinct from antiviral small-molecule drugs. It is essentially a pan-serotype humanized antibody against DENV, capable of binding to the EDIII-exposed areas of the four DENV serotypes. In mouse models of primary infection or secondary fatal antibody infection, VIS513 rapidly reduces the virus titer, prevents fatal infection, and shows low propensity for viral escape in DENV1 and DENV2, although delayed resistance emergence was observed for DENV3 and DENV4 *in vitro* (33). Nevertheless, to ensure optimal safety, it is imperative to optimize dose selection in future clinical trials, which will facilitate the mitigation of potential ADE risk at sub-neutralizing antibody concentrations.

In summary, there are currently 7 main types of dengue vaccines (**Table 1**). Although classic attenuated or inactivated vaccines have low development costs and mature technologies, they mainly induce B-cell responses and variable T-cell immune responses. Therefore, they generally show limited T cell immune responses against non-structural antigens of DENV, which may contribute to immune imbalances against the four DENV serotypes, potentially increasing the risk of secondary infections leading to severe dengue fever by ADE (19). The new vaccine pays more attention to the shortcomings of traditional DENV vaccines, shifting the focus of development to inducing T cell responses while mitigating ADE risks. For example, TV003 induces both CD $8^{+}$ T cell and antibody responses, demonstrating that existing platforms can elicit T cell immunity (17). However, targeted T cell epitope vaccines aim to optimize this response. Additionally, DDV avoids immune interference between serotypes in preclinical models (36). In particular, preclinical studies suggest that mRNA-LNP modified vaccines have attracted much attention due to their potential for rapid, inexpensive, and large-scale production. In mouse models, they show reduced induction of cross-reactive antibodies associated with DENV enhancement, and elicit serotype-specific immunity and T cell responses (28). In these animal models, they resist lethal DENV attacks and represent promising candidates for treating and preventing DENV. However, vaccine design must balance T cell immunogenicity and safety, since excessive T cell responses can drive severe cytokine storms in dengue.

**Table 1:** Current status and dilemmas of dengue vaccine research.

Vaccine type	Vaccine name	Developer	Strategy	Research subject	Major adverse reactions	Key clinical outcome	Vaccine immunogenicity and efficacy		Current stage/ references
Live attenuated vaccines	CYD-TDV	Sanofi Pasteur	YFV-17D backbone	Aged 2-16; Dengue epidemic areas in Asia Pacific, Latin America, and Thailand	Year 3: pooled RR of hospitalization 1.58 (95% CI, 0.83–3.02) in<9 years; 12 severe dengue cases in vaccine group vs. 0 in control group among<9 years	All hospitalized cases have recovered; ≥ 9 years old for stable protection	Symptomatic VCD: total 60.3%, 65.6% for ages ≥ 9, 44.6% for ages<9; Hospitalization: 80.8% for ages ≥ 9, 56.1% for ages<9; Critical care: 93.2% for ages ≥ 9, 44.5% for ages<9; MN: Baseline seronegative 70%, seropositive 82%		Licensed (13, 15, 34)
	TAK-003	Takeda	DENV-2 backbone	4–16 years old; 8 dengue endemic countries	No SAEs	Continued benefit for 2 years regardless of baseline serostatus; Disease-modifying effect; Waning efficacy in Year 2, especially in 4–5 years old	Overall VCD 72.7%, hospitalization 89.2%; DENV-2 (90.8%) is optimal; The baseline serum MN titers are all<10 (negative for 4 dengue serotypes) and ≥ 10 (positive for at least 1 dengue serotype)		Licensed (19)
Inactivated vaccines	DPIV	WRAIR; GSK;	Four formulations: 1 μg/serotype + Alum/AS01E/AS03B, 4 μg/serotype + Alum; Two doses 4 weeks apart	Healthy adults aged 20-39 (Puerto Rico, 90% with a history of dengue infection)	No SAEs	3 years of safe tolerance; DENV1–3 neutralizing antibodies remained higher than before vaccination, with the highest GMT observed in the 1 μ g+AS03B formula	Not evaluated		Phase I (21)
Recombinant subunit vaccines	V180	Merck	DEN-80E +ISCOMATRIX ™/Aluminum hydroxide/no adjuvant;	Healthy flavivirus-naïve adults aged 18–45 years (Australia; N = 98)	No SAEs; ISCOMATRIX™ group: injection-site reactions 98%, fever ≥38 °C 9%, Grade 3 events 21%; Alhydrogel/unadjuvanted: milder reactions	High dose ISCOMATRIX ™ Adjuvant formulations can induce specific memory B cell responses to type 4 dengue virus, increasing by 1–2 logarithmic orders of magnitude compared to baseline, with balanced distribution of various serotypes	SCR ≥85.7% for all 4 serotypes, durable NAb for DENV-1–3 at 1 year (DENV-4 returned to baseline); robust memory B-cell responses (1–2 log increase); Alhydrogel/unadjuvanted formulations poorly immunogenic (SCR 14.3–62.5%, titers waned by 6 months)		Phase I; Standalone development discontinued; (23, 24)
DNA vaccines	D1ME100	Naval Medical Research Centre	Plasmid pVR1012 containing DENV-1 prM and full-length E genes under CMV promoter	Adult male and female Aotus nancymae monkeys (N = 11)	No SAEs	durable neutralizing antibodies and immunologic memory demonstrated; post-challenge anamnestic responses in all vaccines	33% (2/6) completely protected from viremia; 83% (5/6) with complete or partial protection; mean viremia reduced to 1 day (ID) and 1.3 days (IM) vs 4 days (controls); ID route induced stronger and more uniform antibody responses than IM; PRNT50 titers up to >360 (ID) vs 160 (IM) by month 6;		Preclinical; subsequent human trial conducted Phase I (27, 35)
mRNA-modified vaccines	mRNA-LNP Vaccine	University of Illinois College of Medicine, St. Louis University College of Medicine	Pseudouridine modified prM/E mRNA+LNP delivery	C57BL/6J mice, AG129 immunodeficient mice	No SAEs	100% protection in mouse model, low ADE risk	Inducing DENV1 specific neutralizing antibodies (EC50 highest 1/3125) and CD4 ^+^/CD8 ^+^ T cells; 100% survival of AG129 mice after lethal attack; ADE risk is extremely low (only 1.2 times)		Preclinical stage (28)
Adenovirus vector vaccines	CAdVax-DenTV	US Naval Medical Research Center, GenPhar Corporation, etc	Composite adenovirus vector (E1/E4 deletion) containing DENV1–4 prM/E gene	3-8-year-old rhesus monkey (N = 47), dengue fever serum negative	No SAEs	Significant protection against quadrivalent dengue fever in rhesus monkeys		After the second vaccination, quadrivalent neutralizing antibodies (PRNT_50_ mean 200-937) were produced; Attack after 4/24 weeks, DENV1/3 completely viremia free, DENV2/4 viremia significantly shortened	Preclinical stage (29)
Multivalent synthetic peptide vaccines	PepGNP	Unisant é, Switzerland CHUV; Emergex Vaccines funding	9 types of DENV peptides+gold nanoparticles	Healthy adults aged 18-45 (N = 26)	No SAEs	No significant abnormalities in laboratory indicators		AIM CD $8^{+}$T cells: LD group:67%; HD group:10%; Not inducing anti DENV antibodies; The LD group significantly induced CD $8^{+}$T cells and T_cm_/T_em_RA memory cells, while the HD group showed weak response	Phase I (31)

## 3 MHC, HLA, and T cell epitopes

The aforementioned research into dengue vaccines and other therapeutic approaches shows that the main issue affecting the safety and efficacy of dengue vaccines is the ADE phenomenon triggered by a secondary dengue virus infection. Inducing T cell immunity through epitope peptides represents a potential strategy that may help attenuate ADE - related risks, primarily based on preclinical models (37).

### 3.1 Introduction of MHC and HLA

The MHC plays a key role in the recognition of antigen peptides by T cells, which is a group of gene complexes that encode major histocompatibility antigens. These antigens have an influence on allograft rejection, immune regulation, and infectious diseases (38). The human MHC, also known as the human leukocyte antigen (HLA) system, can be divided into three categories, and only the classic class I and class II HLA have high polymorphism and can participate in antigen presentation. HLA class I molecules, which are expressed on the surface of most nucleated cells, mainly recognize and present endogenous antigenic peptides and bind to CD $8^{+}$ T cells, thereby limiting the recognition of cytotoxic T cells. In the context of DENV infection, HLA class I molecules present DENV - derived antigenic peptides, activating specific CD8^+^ T cells implicated in both viral control and immunopathology, including T cell-mediated cytokine storms associated with severe dengue (39). However, certain HLA class I alleles, such as HLA-A*24 and HLA-B*51, have been associated with increased susceptibility to severe dengue, likely due to suboptimal antigen presentation or altered T cell activation that may contribute to immunopathology rather than protection (40, 41). It is noteworthy that HLA is also polymorphic. Classical HLA molecules alone have thousands of alleles and are increasing, which means that they can combine with different antigenic peptides to induce variable immune responses depending on the specific HLA - peptide combination and T cell context (42). Each HLA variant also has different expression frequencies in different races and geographical regions. Therefore, there are individual and regional differences in the reactivity and susceptibility of DENV - infected hosts (43). For example, the high prevalence of HLA-A24 in Southeast Asian populations correlates with the higher burden of severe dengue in this region compared to Latin America, where HLA-A02 is more prevalent, and disease severity patterns differ. However, current DENV T cell epitope studies predominantly focus on HLA-A24:02 and HLA-A11:01, with limited evaluation of population coverage across diverse HLA alleles (40, 43). Consequently, T cell epitope-based vaccines for DENV should incorporate a broad repertoire of antigenic peptides with extensive HLA coverage and population-specific optimization to ensure efficacy across diverse populations and geographical regions, though the feasibility of achieving comprehensive HLA coverage remains a significant challenge.

### 3.2 T cell epitope

**Figure 3:**
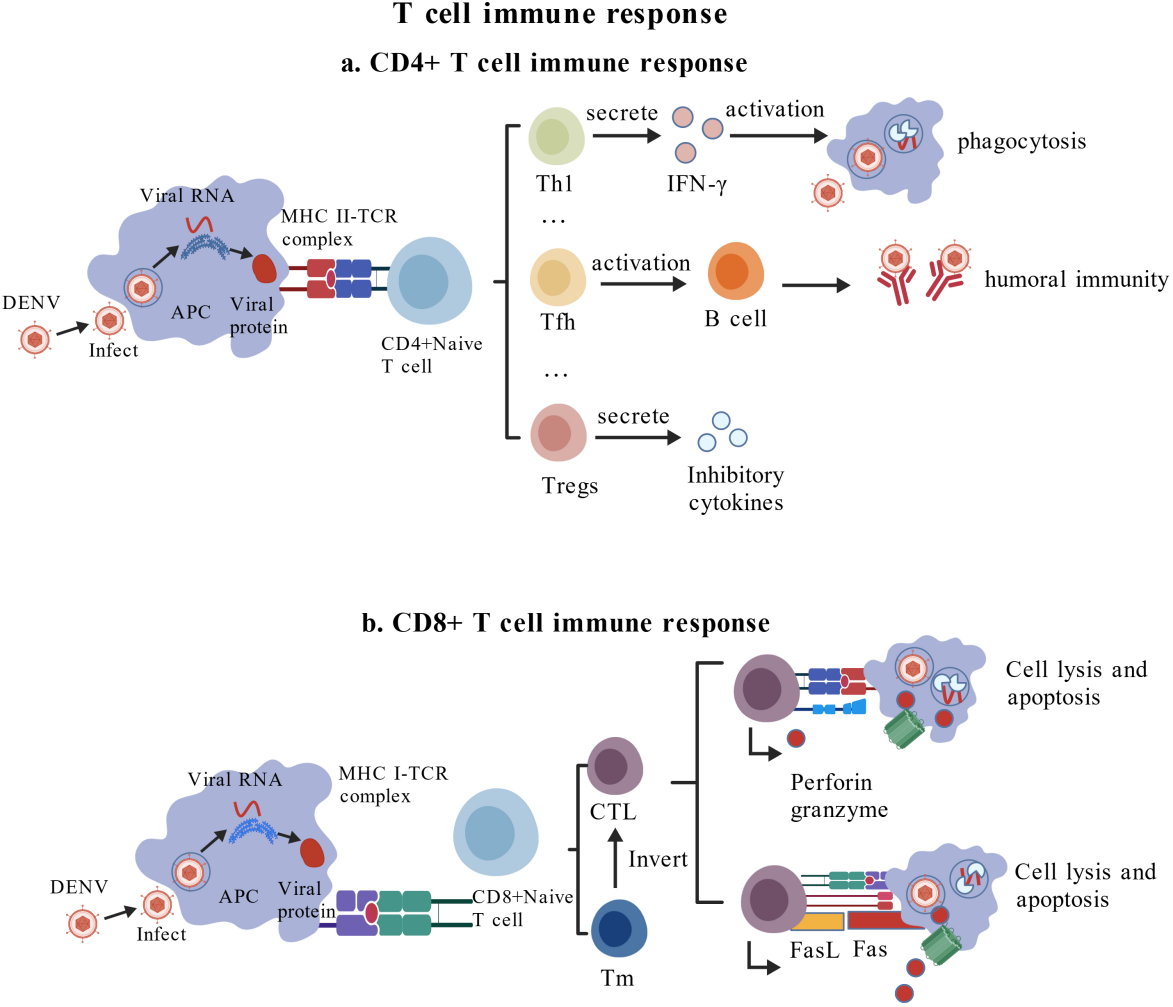
T cell immune response: **(a)** CD $4^{+}$ T cell immune response: when T cell epitopes are successfully recognized, CD $4^{+}$ initial T cells can differentiate into Th1, Tfh, Tregs, and other cells that activate macrophages, promote B cell maturation, induce humoral immunity, or secrete inhibitory cytokines to affect immune responses; **(b)** CD $8^{+}$T cell immune response: when T cell epitopes are successfully recognized, CD $8^{+}$ initial T cells mainly differentiate into cytotoxic T cells (CTLs), which can cleave and apoptotic target cells by secreting perforin or granzyme.

The polymorphism of MHC determines that the T cell epitopes also have their characteristics, expanding the range of antigen peptide presentation in the population. Consequently, this expands the range of antigen peptide presentation of the population, which is conducive to the survival and continuity of the race. Among them, the T cell epitope is a ligand that specifically binds to MHC (**Figure 3**). After being processed by APC, it binds to MHC molecules to form the antigen-peptide-MHC (pMHC) complex, which is recognized by the TCR. Essentially, a linear antigen epitope is formed from the polypeptide fragment, resulting from the degradation of viral structural or non-structural proteins, which are found at any point of the antigen molecule and recognized by CD $4^{+}$T cells and CD $8^{+}$T cells. Recognized by CD $8^{+}$T cells, the epitopes are usually peptides with a length of 8–12 amino acids. The epitopes, which are recognized by CD $4^{+}$T cells, are peptides with a length of 12–25 amino acids. Furthermore, the binding of different peptide segments to their respective HLA alleles is determined by the amino acid at the anchor position. This is typically the second amino acid in the C-terminal region of HLA class I molecules that present peptides (44). In contrast, the binding groove of HLA class II molecules is open at both ends. Thus, different HLA class II molecules exhibit greater inclusivity in terms of peptide length and hybrid peptide binding. When the T cell epitope is successfully recognized, it activates the corresponding T cell proliferation to form a specific effector T cell population. For example, CD $4^{+}$ initial T cells differentiate into Th1, Tfh, Tregs, and other cells, which activate macrophages, promote B cell maturation, and induce humoral immunity. They also secrete inhibitory cytokines that influence the immune response. CD $8^{+}$ initial T cells differentiate mainly into cytotoxic T cells (CTLs) that cleave and apoptose target cells by secreting perforin or granzyme. Some T cell populations detect the same epitope on other cells in the body and quickly form memory T cell populations so that the host can quickly respond and clear the corresponding virus when facing the re-stimulation of the same epitope (45). At present, a large number of CD $4^{+}$ and CD $8^{+}$T cell epitopes have been identified on a variety of DENV proteins, mainly on non-structural proteins (46). A large number of studies on DENV generally believe that CD $8^{+}$T cells preferentially target NS3 and NS5 proteins, while CD $4^{+}$T cells preferentially target E, C, and NS1 proteins (47). In addition, some experimental results showed that the immune-dominant hierarchical structure between structural and non-structural proteins of DENV varied with different serotypes of DENV infection. Specifically, DENV-1, -2, and -4 primarily target CD $8^{+}$T cell responses to non-structural proteins NS3, NS4B, and NS5, while DENV-3 triggers CD $8^{+}$T cell responses specific to structural proteins (C, M, E) and non-structural proteins (48). Different epitope-specific T cells circulate continuously in DENV seropositive individuals. After activation, they not only recognize peptides but also recognize DENV-infected cells by secreting pro-inflammatory cytokines and cytotoxicity (49). However, it is important to note that excessive or dysregulated T cell activation can contribute to immunopathology, including cytokine storms associated with severe dengue hemorrhagic fever (50, 51). The balance between protective and pathogenic T cell responses is influenced by factors such as epitope specificity, HLA restriction, and prior infection history. In particular, numerous studies have confirmed that certain viruses can evade humoral immune responses, such as SARS-CoV-2, HIV, and RNA viruses represented by influenza viruses. This is due to the poor stability of humoral immunity, which makes epitope mutations more likely to occur, significantly limiting the effectiveness of humoral immune responses (52, 53). Cellular immunity offers potential advantages in resisting viral infection and eliminating intracellular pathogens, and may reduce the risk of ADE under certain conditions. Nevertheless, optimal protection likely requires a coordinated response involving both high-quality neutralizing antibodies and appropriately regulated T cell immunity (44). Therefore, whether it can stimulate strong CD $8^{+}$T cell immunity has become a new direction and evaluation standard for many vaccines under development.

### 3.3 T cell immunity to dengue virus

Humoral immunity is associated with the ADE phenomenon, which leads to severe dengue fever and poses challenges for dengue vaccine development. Consequently, studies have shifted the focus to cellular immunity, hoping to complement humoral responses and potentially mitigate ADE-associated risks through robust cellular immunity, a hypothesis supported primarily by preclinical studies, though its clinical relevance remains to be fully established. In some mouse models, CD $8^{+}$T cells demonstrate protective effects on mice infected with homotypic or heterotypic DENV serotypes (54, 55), reducing the risk of antibody-mediated severe dengue fever. At the same time, a cohort study of children with a history of DENV infection in Nicaragua showed that robust CD $8^{+}$T cell-induced T cell immunity was more common in children who had experienced two or more DENV infections and correlated with reduced symptoms upon reinfection (6). In addition, when activated in DENV patients, CD $8^{+}$T cells produce a large number of cytokines such as IFN-γ and TNF, which in turn regulate the activation, growth, and differentiation of cytotoxic T cells. Therefore, CD $8^{+}$T cells directly kill cells with specific antigens or corresponding target cells and show cytotoxic effects. Conserved T cell epitopes may provide targets for broadly reactive vaccines (**Table 2**).

**Table 2:** T cell epitopes of DENV.

Protein type	Target protein	Sequence	Serotype	Host	MHC restriction epitopes	Peptide length	Induced T cell type	Epitopes explanation	References
Structural proteins	prM	AYTIGTTHF	DENV-2	HLA-A * 2402 transgenic mice	HLA-I (HLA-A*2402)	9aa (M 141–149)	CD $8^{+}$	Highly conservative within DENV-2; HLA-A * 2402 has strong restriction and induces CD8 ^+^ T cells to secrete IFN - γ/IL-6, which can kill infected cells	(56)
	E	RHVLGRLITVNPIVT	DENV-2	C57BL/6J mice		15aa (E 345-359)		Located in the E protein EDIII (critical domain for viral invasion); Simultaneously possessing B/T cell epitope activity, inducing IFN - γ secretion (15-45/1 × 10 ^5^ cells), low ADE risk	(57)
		PTLDIELLK	DENV-1	HLA transgenic mice	HLA-I (HLA-A*1101)	9aa (E39-47)	CD $8^{+}$	Secretion: Elispot method: IFN-γ=154 ± 28 SFCs/ ${2*10}^{5}$ SMCs; Peripheral blood:9 ± 1 SFCs/1 × $10^{5}$PBMCs; DENV-1 is highly conservative; HLA-A * 1101 has high affinity and induces CD8 ^+^ T cells to secrete IFN - γ, killing infected cells, which has been validated by human PBMCs	(58)
Structural proteins	C	KLVMAFIAFLRFL	DENV-1; DENV-3	Convalescent patients with DF	HLA-II (DP4,DPw4,DQ1,DQ5,DQ7)	13aa (C45-57)	CD $4^{+}$	ELISPOT detection: IFN - γ secretion level of 50–310 SFC/1 × 10 ^6^ PBMC; ICS detection: IFN - γ ^+^ CD4 ^+^ T cells account for 0.03-0.27% (total CD4 ^+^ T cells); Cross DENV-1/3 conservative; Adapting to multiple HLA-II subtypes, inducing CD4 ^+^ T cells to secrete IFN - γ, validated by PBMCs from recovered patients	(59)
Non-structural proteins	NS1	RGPSIRTTTA	DENV-1/2/3/4	Balb/c mice	MHC-I(H2-D^d^)	10aa (1068–1077 in the polyprotein)	CD $4^{+}$	Conserved across four serotypes; MHC-I (H-2D^d^) binding preferentially induces CD4 ^+^ T cells to secrete IFN - γ without species cross reactivity	(60)
Non-structural proteins	NS2A	TYLALLAAF	DENV-2	HLA-A * 2402 transgenic mice	HLA-I (HLA-A*2402)	9aa (NS2A 73–81)	CD $8^{+}$	Highly conservative within DENV-2; HLA-A * 2402 has strong restriction and induces CD8 ^+^ T cells to secrete IFN - γ/IL-6, which can kill infected cells	(56)
	NS2B	SILLSSLLK	DENV-1	HLA transgenic mice	HLA-1 (HLA-A*1101)	9aa (NS2B 15-23)	CD $8^{+}$	Secretion: Elispot method: IFN-γ=82 ± 22 SFCs/2 × $10^{5}$ SMCs; DENV-1 is highly conservative; HLA-A * 1101 has high affinity and induces CD8 ^+^ T cells to secrete IFN - γ, killing infected cells	(58)
	NS3	SYKVASEGF	DENV-1	HLA transgenic mice	HLA-I (HLA-A*2402)	9aa (NS3 548-556)	CD $8^{+}$	Secretion: Elispot method: IFN-γ=52 ± 12 SFCs/ $2*10^{5}$SMCs; Peripheral blood:11 ± 2 SFCs/1 × $10^{5}$PBMCs DENV-1 is highly conservative; HLA-A * 1101 has high affinity and induces CD8 ^+^ T cells to secrete IFN - γ, killing infected cells, which has been validated by human PBMCs	(58)
Non-structural proteins	NS4A	AYRHAMEEL	DENV-1	HLA transgenic mice	HLA-I(HLA-A*2402)	9aa (NS4A 40-48)	CD $8^{+}$	Secretion: Elispot method: IFN-γ=96 ± 22 SFCs/ $2*10^{5}$ SMCs; Peripheral blood:17 ± 4 SFCs/1 × $10^{5}$PBMCs; DENV-1 is highly conservative; HLA-A * 2402 has high affinity and induces CD8 ^+^ T cells to secrete IFN - γ, killing infected cells, which has been validated by human PBMCs	(58)
	NS4B	VVYDAKFEK	DENV-1	HLA transgenic mice	HLA-I(HLA-A*1101)	9aa (NS4B 159-167)	CD $8^{+}$	Secretion: Elispot method: IFN-γ=56 ± 12 SFCs/ $2*10^{5}$ SMCs; Peripheral blood:7 ± 1 SFCs/1 × $10^{5}$PBMCs; DENV-1 is highly conservative; HLA-A * 1101 has high affinity and induces CD8 ^+^ T cells to secrete IFN - γ, killing infected cells, which has been validated by human PBMCs	(58)
	NS5	TYGWNLVKL	DENV-1/2/3/4	Balb/c mice	MHC-I(H-2K^d^)	9aa (2612–2620 in the polyprotein)	CD $4^{+}$	Conserved across four serotypes; MHC-I (H-2K^d^) binding preferentially induces CD4 ^+^ T cells to secrete IFN - γ without species cross reactivity	(60)

However, viewing T cell immunity merely as a protective mechanism overlooks its pathophysiological complexity. T cells play a “double-edged sword” role in dengue virus infection, mediating viral clearance while also potentially driving the progression of severe dengue. Although DENV - specific CD $8^{+}$ T cells can exert protective antiviral effects during the acute phase by recognizing viral epitopes in an HLA class I-restricted manner, releasing cytotoxic molecules such as perforin and granzymes to directly clear infected cells, an excessively activated T cell response can trigger severe immunopathology through the mechanism of OAS (50, 61). Specifically, during secondary infection, memory T cells preferentially expand and target epitopes that cross-react with previously encountered serotypes. These low-affinity, cross-reactive CD $8^{+}$ T cells, although capable of producing high levels of pro-inflammatory cytokines, exhibit significantly impaired cytotoxic function, manifested as reduced secretion of perforin and granzyme B and decreased degranulation capability, leading to delayed viral clearance coexisting with persistent inflammation (50, 62). This dysfunctional T cell response further drives the activation of monocytes, macrophages, and endothelial cells, generating a positive feedback cytokine cascade, popularly known as a “cytokine storm, “ characterized by a sharp increase in plasma levels of TNF-α, IL-6, IL-8, IL-10, and IFN-γ, directly inducing endothelial cell apoptosis, degradation of tight junction proteins, and increased vascular permeability, ultimately causing plasma leakage, hemorrhagic tendencies, and circulatory failure, which are central to the pathogenesis of DHF/DSS (63). It can be seen that T cell immunity helps regulate the disease, but inactivated immunity against DENV fundamentally still requires the involvement of neutralizing antibodies, and vaccine strategies that rely solely on T cells have shown limited efficacy in clinical trials (64). Therefore, protective immunity against DENV requires a balance between B cell and T cell responses.

### 3.4 Dengue virus T cell epitopes: prediction, population coverage, and vaccine development

Presently, IEDB and other websites have successfully predicted more than 2000 kinds of “dengue virus”, “positive”, “human-derived”, and “T cell” epitope peptides. Some studies have constructed peptides corresponding to these to immunize mice for immune response determination. According to the ELISPOT method, Intracellular Cytokine Staining (ICS) method, and other experimental results, it is not difficult to find that most T cell epitopes induce CD $8^{+}$T cells or CD $4^{+}$T cells to secrete high levels of IFN-γ (60, 65). In addition, an anti-EDIII antibody is generally considered a strong neutralizing antibody and more virus-type specific (66). Among them, T cell epitope peptides E 345–359 and B cell epitope peptide E 383–397 from DENV-2 (NGC strain) EDIII significantly react with mouse anti-DENV-2 (NGC strain) serum, ascites, and sera of patients with dengue fever recovery. ELISPOT test results also showed that E 345–359 induced the secretion of higher IFN-γ, which can be used to evaluate the effect of humoral and cellular immunity in mice and the specific diagnosis of DENV infection (57). Further research has confirmed that the strong immune response induced by multifunctional CD $8^{+}$T cells has a gratifying protective effect against related diseases caused by DENV.

Given the high frequency of HLA-A*2402 and HLA-A*1101 alleles in East Asian populations, Duan et al. subsequently examined the HLA restriction of predicted peptides in HLA-A*2402 and HLA-A*1101 transgenic mice, through IFN-γ ELISPOT assays, and other experiments confirmed the specific T cell immune responses induced by corresponding T cell epitope peptides (56). A series of experimental results demonstrated that multiple binding peptides derived from DENV-2 significantly induced IFN-γ-secreting T cell immune responses, including E 298–306, M 141–149, NS2A 73–81, NS4B 228–237, and NS5 475–484. M 141–149 and NS5 475–484 additionally induced IL-6 production, whilst E 298–306, NS2A 73–81, and NS4B 228–237 triggered IL-6 and TNF-α production by multifunctional CD8^+^ T cells (56). While these responses were characterized in HLA-A*24:02 and HLA-A*11:01 contexts, these alleles only represent a small portion of global HLA diversity. Population coverage analysis shows that a reference set containing 27 HLA class I alleles overall covers more than 97% of the global population, but there are significant regional differences. HLA-A24:02 is more common in Southeast Asian populations, while HLA-A02:01 predominates in Caucasian populations (67, 68). Therefore, to achieve broad population coverage, it is necessary to include multiple HLA allele-restricted epitopes. In addition, Sánchez-Burgos et al. used computational technology to predict and analyze 21 potential DENV T cell epitopes. Most of these T cell epitopes are located in proteins NS5 and E and can induce the activation of specific antibodies or T cells. Moreover, 13 epitope peptides showed immunogenicity in mouse experiments (60). Notably, these immunogenic epitopes were predicted to bind multiple HLA class I supertypes (HLA-A2, A3, B7, B44), suggesting potential broad population coverage (69). However, the dengue DNA vaccine targeting the EDIII-NS1 consensus sequence and the mRNA LNP vaccine encoding the prM-E gene both induced broad-spectrum T cell responses and neutralizing antibodies against four serotypes in preclinical studies in mice, without triggering ADE-related cross reactions. However, both types of vaccines are still in the preclinical stage, and their protective efficacy based on T cell epitopes and ADE prevention effect still need to be validated through human clinical trials (37).

## 4 T cell epitope induced cellular immunity in avoiding ADE

### 4.1 T cell epitope vaccine

Due to the significant protective effect of the T cell response induced by T cell epitopes, many of the DENV vaccines demonstrate the importance of T cell epitopes in inducing cellular immunity and potentially contributing to the reduction of ADE risk to varying degrees (9, 70). In the process of developing live attenuated vaccines, individuals who have previously been infected with DENV and received live attenuated tetravalent DENV demonstrate T cell responses skewed toward conserved epitopes, with non-structural proteins representing the dominant targets of CD $8^{+}$ T cell responses (71). Nevertheless, the DENV-1 vaccine contains structural and non-structural proteins, which induce multifunctional T cell responses. Lindow et al. found that individuals with low viral load usually have a high proportion of multifunctional T cells, especially IFN-γ+TNF-α+IL-2 (triple producer) or IFN-γ+IL-2 (double producer) CD $4^{+}$T cells. Individuals with high viral load and severe disease progression will produce more IFN-γ+CD $4^{+}$T cells. Thus, high levels of pro-inflammatory cytokines may appear to help control and clear viral infections (72). Furthermore, the phenomenon of OAS at the T cell level, where sequential DENV infections may skew T cell memory toward previously encountered serotypes, represents an important consideration for T cell epitope vaccine design (50). Chawla et al. have compared six candidate vaccines with the DENV T cell epitopes of four serotypes. The results showed that almost half of the known CD $4^{+}$T cell epitopes of DENV 1, DENV 3, and DENV 4 existed in TV003, TDEN, and DPIV, while in DENGVAXIA^®^, DENVax and TVDV account for less than one-tenth (73). In DENV-2, CD $4^{+}$T cell epitopes account for a large proportion of TDEN, DPIV, and DENVax, while the proportion of CD $4^{+}$T cell epitopes in the other three vaccines is relatively small, which may be related to the coding or destruction and replacement of non-structural components of DENV. DENGVAXIA^®^, LAV-TDV, and TAK-003 are three well-known major anti-DENV vaccines. According to Ramos Pinheiro’s conservative epitope analysis, it was found that 29 T cell epitopes were not conserved in the three vaccine antigens, and 2 T cell epitopes were not preserved in the vaccine or the circulating virus. Compared with TAK-003 and LAV-TDV vaccines, DENGVAXIA^®^ skeleton mainly lacks a large number of non-structural proteins and capsid proteins targeting DENV T cell epitopes, and the risk of producing ADE in animal experiments and clinical trials is much higher than that of the latter two vaccines (19). DENV1-NS, a minimal DENV antigen designed from a conserved T cell epitope, contains four regions rich in CD $8^{+}$ T cell epitopes. This triggers a strong CD $8^{+}$ T cell response, successfully reducing the incidence and mortality of viremia in transgenic mice and delaying the onset of disease. Generally, it is not difficult to find that DENV’s CD $8^{+}$T cells preferentially target NS3 and NS5 proteins, while CD $4^{+}$T cells preferentially target E, C, and NS1 proteins. Therefore, the DENV vaccine containing more conservative T cell epitopes (especially CD $8^{+}$T cells) will be more effective in preventing and treating viral infection while potentially reducing the risk of ADE (47). However, optimal protection likely requires a balanced immune response incorporating both high-quality neutralizing antibodies and robust T cell immunity, rather than relying solely on T cell responses.

### 4.2 DENV mRNA-LNP vaccine

T cell epitopes have emerged as a promising strategy to enhance protective immunity against DENV and other flaviviruses, with the potential to mitigate the ADE risks associated with antibody-centric vaccines. Preclinical studies have demonstrated that engineering DENV proteins to optimize T cell epitope presentation can elicit robust and specific cellular immune responses. However, the protective efficacy of T cell-centric vaccination strategies in humans remains to be fully validated, as T cell responses may also contribute to immunopathological damage under certain contexts. Therefore, balanced vaccine designs that integrate optimally selected T cell epitopes with the induction of high-quality neutralizing antibody responses may represent a more effective approach to address the long-standing challenges in DENV vaccine development (74). However, whether this serotype-specificity translates to reduced ADE risk in humans remains to be determined. Similarly, Zhang et al. designed prME/E/NS1 mRNA vaccines that encode DENV-2 strain T cell epitopes and encapsulated in LNP. These mRNA LNP vaccines induce antigen-specific immune responses in BALB/c mice (10). The E-mRNA vaccine and NS1 mRNA vaccine also induce the production of strong neutralizing antibodies and T cell immune responses, and provide comprehensive protection against DENV-2 infection. The NS1 mRNA vaccines that lack neutralizing antibody sites will not be expressed on the surface of the DENV. Therefore, its injection alone or in combination with the E-mRNA vaccine proved effective in preventing DENV-2 infection without inducing neutralizing antibodies, thereby significantly reducing ADE. However, there is currently no strong evidence to confirm that these neutralizing antibodies will not become sub-neutralizing antibodies in clinical practice, and further research is still needed. Claude Roth focused on the potential advantages of T cell epitopes in the treatment and prevention of DENV infection. They designed a minimal DENV antigen DENV1-NS from the CD $8^{+}$T cell epitopes of NS3, NS4B, and NS5 in type I DENV, which can induce a strong CD $8^{+}$T cell response in mouse models. Compared to most existing DENV vaccines, it does not induce the production of antibodies against prM or E proteins, thus decreasing the risk of ADE.

From the above results, it can be seen that the development of DENV mRNA-LNP vaccines is still mainly focused on inducing the DENV’s prM or E proteins. Its advantage lies in simultaneously eliciting both B-cell and T-cell immune responses. Compared with simple B cell immunity, the presence of T cell immunity provides greater resistance to DENV attacks by stimulating antigen-specific CD $4^{+}$ and CD $8^{+}$ T cells, as well as high levels of cytokines such as IFN-γ and TNF-α. This enhances the overall immune protection effect and reduces the production of suboptimal antibodies, alleviating ADE caused by cross-reactivity (9).

## 5 Summary and outlook

In recent years, the number and severity of cases related to DENV have increased (75). In order to effectively control DENV infection, developing safe and effective dengue vaccines is a key measure to prevent and control infection. Currently, the development of DENV vaccines mainly aims to stimulate the B-cell epitopes of DENV to induce high-level antibody neutralization. Although B-cell and T cell responses in humans are typically induced by the dengue virus, leading to effective and long-lasting immunity, it has been indicated by relevant studies that the development of severe dengue fever is actually promoted by secondary infection with the DENV (5). At the same time, these chimeric vaccines lack T cell immune responses against the non-structural antigens of DENV. The efficacy of DENV vaccines against different serotypes is reduced and imbalanced. Therefore, ADE has become one of the driving forces behind the increasing frequency of severe dengue fever, severely restricting the safety and efficacy of dengue vaccines. However, it is important to note that T cell responses are not uniformly protective; uncontrolled T cell activation can contribute to immunopathology through cytokine storm in severe dengue, and the concept of OAS in T cell memory remains insufficiently addressed in current vaccine strategies. The lack of strong DENV-specific T cell responses may also be the main reason for the failure of the efficacy of DENV quadrivalent attenuated chimeric vaccines. Some studies have shown that appropriately regulated T cell immunity may help mitigate ADE between DENV and flavivirus by inducing CD $4^{+}$or CD $8^{+}$T cells and cytokines such as IFN γ and TNF. Moreover, DENV vaccines containing more conserved T cell epitopes may potentially offer advantages in preventing and treating viral infections while weakening ADE risk. If the selected T cell epitope is conserved, it can generate cross-protection against infections from different serotypes, as well as other yellow viruses, providing effective, broad-spectrum protection. In summary, a universal vaccine based on conserved T cell epitopes and capable of inducing broad, specific, multifunctional, and cross-reactive T cell responses in four DENV serotypes represents a promising direction, though clinical validation remains essential to confirm that enhanced T cell responses can indeed improve safety and efficacy without exacerbating immunopathology.

## References

[1] Wolf J Souza AP Schardosim RFC Pille A Maccari JG Mutlaq MP . Correction to: Molecular evolution of dengue virus: a Bayesian approach using 1581 whole-genome sequences from January 1944 to July 2022. Arch Virol. (2023) 168:212. doi: 10.1007/s00705-023-05833-3. PMID: 37518501

[2] Bhatt S Gething PW Brady OJ Messina JP Farlow AW Moyes CL . The global distribution and burden of dengue. Nature. (2013) 496:504–7. doi: 10.1038/nature12060. PMID: 23563266 PMC3651993

[3] Inizan C Minier M Prot M O’Connor O Forfait C Laumond S . Viral evolution sustains a dengue outbreak of enhanced severity. Emerging Microbes Infect. (2021) 10:536–44. doi: 10.1080/22221751.2021.1899057. PMID: 33686914 PMC8011692

[4] Guzman MG Alvarez M Halstead SB . Secondary infection as a risk factor for dengue hemorrhagic fever/dengue shock syndrome: an historical perspective and role of antibody-dependent enhancement of infection. Arch Virol. (2013) 158:1445–59. doi: 10.1007/s00705-013-1645-3. PMID: 23471635

[5] Shukla R Ramasamy V Shanmugam RK Ahuja R Khanna N . Antibody-dependent enhancement: a challenge for developing a safe dengue vaccine. Front Cell Infect Microbiol. (2020) 10:572681. doi: 10.3389/fcimb.2020.572681. PMID: 33194810 PMC7642463

[6] Gálvez RI Martínez-Pérez A Escarrega EA Singh T Zambrana JV Balmaseda Á . Frequency of dengue virus-specific T cells is related to infection outcome in endemic settings. JCI Insight. (2025) 10:e179771. doi: 10.1172/jci.insight.179771. PMID: 39989460 PMC11949061

[7] Sun J Zheng Z Li M Liu Z Su X Jin X . Development of a novel ZIKV vaccine comprised of immunodominant CD4+ and CD8+ T cell epitopes identified through comprehensive epitope mapping in Zika virus infected mice. Vaccine. (2021) 39:5173–86. doi: 10.1016/j.vaccine.2021.07.036. PMID: 34353682

[8] Zellweger RM Eddy WE Tang WW Miller R Shresta S . CD8+ T cells prevent antigen-induced antibody-dependent enhancement of dengue disease in mice. J Immunol (Baltimore Md: 1950). (2014) 193:4117–24. doi: 10.4049/jimmunol.1401597. PMID: 25217165 PMC4185219

[9] He L Sun W Yang L Liu W Li J . A multiple-target mRNA-LNP vaccine induces protective immunity against experimental multi-serotype DENV in mice. Virol Sin. (2022) 37:746–57. doi: 10.1016/j.virs.2022.07.003. PMID: 35835315 PMC9583182

[10] Zhang M Sun J Li M Jin X . Modified mRNA-LNP vaccines confer protection against experimental DENV-2 infection in mice. Mol Ther - Methods Clin Dev. (2020) 18:702–12. doi: 10.1016/j.omtm.2020.07.013. PMID: 32913878 PMC7452130

[11] Vatti A Monsalve DM Pacheco Y Chang C Anaya JM Gershwin ME . Original antigenic sin: a comprehensive review. J Autoimmun. (2017) 83:12–21. doi: 10.1016/j.jaut.2017.04.008. PMID: 28479213

[12] Torres-Flores JM Reyes-Sandoval A Salazar MI . Dengue vaccines: an update. BioDrugs. (2022) 36:325–36. doi: 10.1007/s40259-022-00531-z. PMID: 35608749 PMC9127483

[13] Huang C-H Tsai Y-T Wang S-F Wang W-H Chen Y-H . Dengue vaccine: an update. Expert Rev Anti-infect Ther. (2021) 19:1495–502. doi: 10.1080/14787210.2021.1949983. PMID: 34182875

[14] Tully D Griffiths CL . Dengvaxia: the world’s first vaccine for prevention of secondary dengue. Ther Adv Vaccines Immunother. (2021) 9:25151355211015839. doi: 10.1177/25151355211015839. PMID: 34036241 PMC8132086

[15] Hadinegoro SR Arredondo-García JL Capeding MR Deseda C Chotpitayasunondh T Dietze R . Efficacy and long-term safety of a dengue vaccine in regions of endemic disease. N Engl J Med. (2015) 373:1195–206. doi: 10.1056/nejmoa1506223. PMID: 26214039

[16] Patel SS Winkle P Faccin A Nordio F LeFevre I Tsoukas CG . An open-label, phase 3 trial of TAK-003, a live attenuated dengue tetravalent vaccine, in healthy US adults: immunogenicity and safety when administered during the second half of a 24-month shelf-life. Hum Vaccin Immunother. (2023) 19:2254964. doi: 10.1080/21645515.2023.2254964. PMID: 37846724 PMC10583633

[17] Whitehead SS . Development of TV003/TV005, a single dose, highly immunogenic live attenuated dengue vaccine; what makes this vaccine different from the Sanofi-Pasteur CYD™ vaccine? Expert Rev Vaccines. (2015) 15:509–17. doi: 10.1586/14760584.2016.1115727. PMID: 26559731 PMC4956407

[18] Patel SS Rauscher M Kudela M Pang H . Clinical safety experience of TAK-003 for dengue fever: a new tetravalent live attenuated vaccine candidate. Clin Infect Dis. (2023) 76:e1350–9. doi: 10.1093/cid/ciac418. PMID: 35639602 PMC9907483

[19] López-Medina E Biswal S Saez-Llorens X Borja-Tabora C Bravo L Sirivichayakul C . Efficacy of a dengue vaccine candidate (TAK-003) in healthy children and adolescents 2 years after vaccination. J Infect Dis. (2022) 225:1521–32. doi: 10.1093/infdis/jiaa761. PMID: 33319249 PMC9071282

[20] Rothman AL Ennis FA . Dengue vaccine: the need, the challenges, and progress. J Infect Dis. (2016) 214:825–7. doi: 10.1093/infdis/jiw068. PMID: 26908750 PMC4996144

[21] Diaz C Koren M Lin L Martinez LJ Eckels KH Campos M . Safety and immunogenicity of different formulations of a tetravalent dengue purified inactivated vaccine in healthy adults from Puerto Rico: final results after 3 years of follow-up from a randomized, placebo-controlled phase I study. Am J Trop Med Hyg. (2020) 102:951–4. doi: 10.4269/ajtmh.19-0461. PMID: 32124728 PMC7204593

[22] Sun P Jani V Johnson A Cheng Y Nagabhushana N Williams M . T cell and memory B cell responses in tetravalent DNA, tetravalent inactivated and tetravalent live-attenuated prime-boost dengue vaccines in rhesus macaques. Vaccine. (2021) 39:7510–20. doi: 10.1016/j.vaccine.2021.10.017. PMID: 34823910

[23] Manoff SB Sausser M Falk Russell A Martin J Radley D Hyatt D . Immunogenicity and safety of an investigational tetravalent recombinant subunit vaccine for dengue: results of a phase I randomized clinical trial in flavivirus-naïve adults. Hum Vaccines Immunother. (2019) 15:2195–204. doi: 10.1080/21645515.2018.1546523. PMID: 30427741 PMC6773383

[24] Torresi J Ebert G Pellegrini M . Vaccines licensed and in clinical trials for the prevention of dengue. Hum Vaccines Immunother. (2017) 13:1059–72. doi: 10.1080/21645515.2016.1261770. PMID: 28281864 PMC5443395

[25] Porter KR Teneza-Mora N Raviprakash K . Tetravalent DNA vaccine product as a vaccine candidate against dengue. Methods Mol Biol. (2014) 1143:283–95. doi: 10.1007/978-1-4939-0410-5_17. PMID: 24715294

[26] Porter KR Ewing D Chen L Wu SJ Hayes CG Ferrari M . Immunogenicity and protective efficacy of a vaxfectin-adjuvanted tetravalent dengue DNA vaccine. Vaccine. (2012) 30:336–41. doi: 10.1016/j.vaccine.2011.10.085. PMID: 22085548

[27] Kochel TJ Raviprakash K Hayes CG Watts DM Russell KL Gozalo AS . A dengue virus serotype-1 DNA vaccine induces virus neutralizing antibodies and provides protection from viral challenge in Aotus monkeys. Vaccine. (2000) 18:3166–73. doi: 10.1016/s0264-410x(00)00105-5. PMID: 10856796

[28] Wollner CJ Richner M Hassert MA Pinto AK Brien JD Richner JM . A dengue virus serotype 1 mRNA-LNP vaccine elicits protective immune responses. J Virol. (2021) 95:e02482-20. doi: 10.1128/JVI.02482-20. PMID: 33762420 PMC8315947

[29] Raviprakash K Wang D Ewing D Holman DH Block K Woraratanadharm J . A tetravalent dengue vaccine based on a complex adenovirus vector provides significant protection in rhesus monkeys against all four serotypes of dengue virus. J Virol. (2008) 82:6927–34. doi: 10.1128/jvi.02724-07. PMID: 18480438 PMC2446963

[30] Awakoaiye B Li S Sanchez S Dangi T Irani N Arroyo L . Comparative analysis of adenovirus, mRNA, and protein vaccines reveals context-dependent immunogenicity and efficacy. JCI Insight. (2025) 10:e198069. doi: 10.1172/jci.insight.198069. PMID: 41212056 PMC12643482

[31] Miauton A Audran R Besson J Maby-El Hajjami H Karlen M Warpelin-Decrausaz L . Safety and immunogenicity of a synthetic nanoparticle-based, T cell priming peptide vaccine against dengue in healthy adults in Switzerland: a double-blind, randomized, vehicle-controlled, phase 1 study. EBioMedicine. (2024) 99:104922. doi: 10.1016/j.ebiom.2023.104922. PMID: 38128414 PMC10776924

[32] Kiemel D Kröll AH Denolly S Haselmann U Bonfanti JF Andres JI . Pan-serotype dengue virus inhibitor JNJ-A07 targets NS4A-2K-NS4B interaction with NS2B/NS3 and blocks replication organelle formation. Nat Commun. (2024) 15:6080. doi: 10.1038/s41467-024-50437-3. PMID: 39030239 PMC11271582

[33] Budigi Y Ong EZ Robinson LN Ong LC Rowley KJ Winnett A . Neutralization of antibody-enhanced dengue infection by VIS513, a pan serotype reactive monoclonal antibody targeting domain III of the dengue E protein. PLoS Negl Trop Dis. (2018) 12:e0006209. doi: 10.1371/journal.pntd.0006209. PMID: 29425203 PMC5823465

[34] Carpp LN Fong Y Bonaparte M Moodie Z Juraska M Huang Y . Microneutralization assay titer correlates analysis in two phase 3 trials of the CYD-TDV tetravalent dengue vaccine in Asia and Latin America. PLoS One. (2020) 15:e0234236. doi: 10.1371/journal.pone.0234236. PMID: 32542024 PMC7295445

[35] Beckett CG Tjaden J Burgess T Danko JR Tamminga C Simmons M . Evaluation of a prototype dengue-1 DNA vaccine in a phase 1 clinical trial. Vaccine. (2011) 29:960–8. doi: 10.1016/j.vaccine.2010.11.050. PMID: 21111785

[36] Danko JR Kochel T Teneza-Mora N Luke TC Raviprakash K Sun P . Safety and immunogenicity of a tetravalent dengue DNA vaccine administered with a cationic lipid-based adjuvant in a phase 1 clinical trial. Am J Trop Med Hyg. (2018) 98:849–56. doi: 10.4269/ajtmh.17-0416. PMID: 29363446 PMC5930886

[37] Sankaradoss A Jagtap S Nazir J Moula SE Modak A Fialho J . Immune profile and responses of a novel dengue DNA vaccine encoding an EDIII-NS1 consensus design based on Indo-African sequences. Mol Ther. (2022) 30:2058–77. doi: 10.1016/j.ymthe.2022.01.013. PMID: 34999210 PMC8736276

[38] Shiina T Hosomichi K Inoko H Kulski JK . The HLA genomic loci map: expression, interaction, diversity and disease. J Hum Genet. (2009) 54:15–39. doi: 10.1038/jhg.2008.5. PMID: 19158813

[39] Rothman AL . Immunity to dengue virus: a tale of original antigenic sin and tropical cytokine storms. Nat Rev Immunol. (2011) 11:532–43. doi: 10.1038/nri3014. PMID: 21760609

[40] Nguyen TP Kikuchi M Vu TQ Do QH Tran TT Vo DT . Protective and enhancing HLA alleles, HLA-DRB1*0901 and HLA-A*24, for severe forms of dengue virus infection, dengue hemorrhagic fever and dengue shock syndrome. PloS NeglTrop Dis. (2008) 2:e304. doi: 10.1371/journal.pntd.0000304. PMID: 18827882 PMC2553281

[41] Stephens HA Klaythong R Sirikong M Vaughn DW Green S Kalayanarooj S . HLA-A and -B allele associations with secondary dengue virus infections correlate with disease severity and the infecting viral serotype in ethnic Thais. Tissue Antigens. (2002) 60:309–18. doi: 10.1034/j.1399-0039.2002.600405.x. PMID: 12472660

[42] Molero-Abraham M Lafuente EM Reche P . Customized predictions of peptide-MHC binding and T-cell epitopes using EPIMHC. Methods Mol Biol. (2014) 1184:319–32. doi: 10.1007/978-1-4939-1115-8_18. PMID: 25048133 PMC7122735

[43] Cao K Hollenbach J Shi X Shi W Chopek M Fernández-Viña MA . Analysis of the frequencies of HLA-A, B, and C alleles and haplotypes in the five major ethnic groups of the United States reveals high levels of diversity in these loci and contrasting distribution patterns in these populations. Hum Immunol. (2001) 62:1009–40. doi: 10.1016/s0198-8859(01)00298-1. PMID: 11543903

[44] Schroeder SM Nelde A Walz JS . Viral T-cell epitopes – identification, characterization and clinical application. Semin Immunol. (2023) 66. doi: 10.1016/j.smim.2023.101725. PMID: 36706520

[45] Peters B Nielsen M Sette A . T cell epitope predictions. Annu Rev Immunol. (2020) 38:123–45. doi: 10.1146/annurev-immunol-082119-124838. PMID: 32045313 PMC10878398

[46] Mathew A Townsley E Ennis FA . Elucidating the role of T cells in protection against and pathogenesis of dengue virus infections. Future Microbiol. (2014) 9:411–25. doi: 10.2217/fmb.13.171. PMID: 24762312 PMC4113002

[47] Rivino L Kumaran EA Jovanovic V Nadua K Teo EW Pang SW . Differential targeting of viral components by CD4+ versus CD8+ T lymphocytes in dengue virus infection. J Virol. (2013) 87:2693–706. doi: 10.1128/jvi.02675-12. PMID: 23255803 PMC3571409

[48] Weiskopf D Angelo MA Sidney J Peters B Shresta S Sette A . Immunodominance changes as a function of the infecting dengue virus serotype and primary versus secondary infection. J Virol. (2014) 88:11383–94. doi: 10.1128/jvi.01108-14. PMID: 25056881 PMC4178794

[49] Comber JD Karabudak A Huang X Piazza PA Marques ET Philip R . Dengue virus specific dual HLA binding T cell epitopes induce CD8+ T cell responses in seropositive individuals. Hum Vaccin Immunother. (2014) 10:3531–43. doi: 10.4161/21645515.2014.980210. PMID: 25668665 PMC4514071

[50] Mongkolsapaya J Dejnirattisai W Xu XN Vasanawathana S Tangthawornchaikul N Chairunsri A . Original antigenic sin and apoptosis in the pathogenesis of dengue hemorrhagic fever. Nat Med. (2003) 9:921–7. doi: 10.1038/nm887. PMID: 12808447

[51] Srikiatkhachorn A Mathew A Rothman AL . Immune-mediated cytokine storm and its role in severe dengue. Semin Immunopathol. (2017) 39:563–74. doi: 10.1007/s00281-017-0625-1. PMID: 28401256 PMC5496927

[52] Stefic K Bouvin-Pley M Braibant M Barin F . Impact of HIV-1 diversity on its sensitivity to neutralization. Vaccines. (2019) 7:74. doi: 10.3390/vaccines7030074. PMID: 31349655 PMC6789624

[53] Caniels TG Bontjer I van der Straten K Poniman M Burger JA Appelman B . Emerging SARS-CoV-2 variants of concern evade humoral immune responses from infection and vaccination. Sci Adv. (2021) 7:eabj5365. doi: 10.1126/sciadv.abj5365. PMID: 34516917 PMC8442901

[54] Yauch LE Zellweger RM Kotturi MF Qutubuddin A Sidney J Peters B . A protective role for dengue virus-specific CD8+ T cells. J Immunol (Baltimore Md: 1950). (2009) 182:4865–73. doi: 10.4049/jimmunol.0801974. PMID: 19342665 PMC2674070

[55] Halstead SB . Dengue. Lancet (London England). (2007) 370:1644–52. doi: 10.1016/b978-0-323-82763-8.00184-9. PMID: 17993365

[56] Duan ZL Liu HF Huang X Wang SN Yang JL Chen XY . Identification of conserved and HLA-A*2402-restricted epitopes in dengue virus serotype 2. Virus Res. (2015) 196:5–12. doi: 10.1016/j.virusres.2014.10.022. PMID: 25449574

[57] Li S Peng L Zhao W Zhong H Zhang F Yan Z . Synthetic peptides containing B- and T-cell epitope of dengue virus-2 E domain III provoked B- and T-cell responses. Vaccine. (2011) 29:3695–702. doi: 10.1016/j.vaccine.2011.03.002. PMID: 21419774

[58] Duan Z Guo J Huang X Liu H Chen X Jiang M . Identification of cytotoxic T lymphocyte epitopes in dengue virus serotype 1. J Med Virol. (2015) 87:1077–89. doi: 10.1002/jmv.24167. PMID: 25777343

[59] Sánchez-Burgos G Ramos-Castañeda J Cedillo-Rivera R Dumonteil E . Immunogenicity of novel dengue virus epitopes identified by bioinformatic analysis. Virus Res. (2010) 153:113–20. doi: 10.1016/j.virusres.2010.07.014. PMID: 20638434

[60] Rouers A Chng MHY Lee B Rajapakse MP Kaur K Toh YX . Immune cell phenotypes associated with disease severity and long-term neutralizing antibody titers after natural dengue virus infection. Cell Rep Med. (2021) 2:100278. doi: 10.1016/j.xcrm.2021.100278. PMID: 34095880 PMC8149372

[61] Srikiatkhachorn A . Plasma leakage in dengue haemorrhagic fever. Thromb Haemostasis. (2009) 102:1042–9. doi: 10.1160/th09-03-0208. PMID: 19967133 PMC5527705

[62] Khanam A Gutiérrez-Barbosa H Lyke KE Chua JV . Immune-mediated pathogenesis in dengue virus infection. Viruses. (2022) 14:2575. doi: 10.3390/v14112575. PMID: 36423184 PMC9699586

[63] Ooi EE Kalimuddin S . Insights into dengue immunity from vaccine trials. Sci Transl Med. (2023) 15:eadh3067. doi: 10.1126/scitranslmed.adh3067. PMID: 37437017

[64] Tian Y Grifoni A Sette A Weiskopf D . Human T cell response to dengue virus infection. Front Immunol. (2019) 10:2125. doi: 10.3389/fimmu.2019.02125. PMID: 31552052 PMC6737489

[65] Chávez JH Silva JR Amarilla AA Moraes Figueiredo LT . Domain III peptides from flavivirus envelope protein are useful antigens for serologic diagnosis and targets for immunization. Biologicals. (2010) 38:613–8. doi: 10.1016/j.biologicals.2010.07.004. PMID: 20817489

[66] Bui HH Sidney J Li W Fusseder N Sette A . Development of an epitope conservancy analysis tool to facilitate the design of epitope-based diagnostics and vaccines. BMC Bioinf. (2007) 8:361. doi: 10.1186/1471-2105-8-361. PMID: 17897458 PMC2233646

[67] Gonzalez-Galarza FF McCabe A Santos E Jones J Takeshita L Ortega-Rivera ND . Allele frequency net database (AFND) 2020 update: gold-standard data classification, open access genotype data and new query tools. Nucleic Acids Res. (2020) 48:D783–8. doi: 10.1093/nar/gkz1029. PMID: 31722398 PMC7145554

[68] Zhang GL DeLuca DS Keskin DB Chitkushev L Zlateva T Lund O . MULTIPRED2: a computational system for large-scale identification of peptides predicted to bind to HLA supertypes and alleles. J Immunol Methods. (2011) 374:53–61. doi: 10.1016/j.jim.2010.11.009. PMID: 21130094 PMC3090484

[69] Roth C Cantaert T Colas C Prot M Casadémont I Levillayer L . A modified mRNA vaccine targeting immunodominant NS epitopes protects against dengue virus infection in HLA class I transgenic mice. Front Immunol. (2019) 10:1424. doi: 10.3389/fimmu.2019.01424. PMID: 31293584 PMC6598640

[70] Ngono AE Shresta S . Immune response to dengue and Zika. Annu Rev Immunol. (2018) 36:279–308. doi: 10.1146/annurev-immunol-042617-053142. PMID: 29345964 PMC5910217

[71] Lindow JC Borochoff-Porte N Durbin AP Whitehead SS Fimlaid KA Bunn JY . Primary vaccination with low dose live dengue 1 virus generates a proinflammatory, multifunctional T cell response in humans. PloS NeglTrop Dis. (2012) 6:e1742. doi: 10.1371/journal.pntd.0001742. PMID: 22816004 PMC3398956

[72] Chawla YM Bajpai P Saini K Reddy ES Patel AK Murali-Krishna K . Regional variation of the CD4 and CD8 T cell epitopes conserved in circulating dengue viruses and shared with potential vaccine candidates. Viruses. (2024) 16:730. doi: 10.3390/v16050730. PMID: 38793612 PMC11126086

[73] Wollner CJ Richner M Hassert MA Pinto AK Brien JD Richner JM . A dengue virus serotype 1 mRNA-LNP vaccine elicits protective immune responses. J Virol. (2021) 95:e02482-20. doi: 10.1128/JVI.02482-20. PMID: 33762420 PMC8315947

[74] Santos LLM de Aquino EC Fernandes SM Ternes YMF Feres VCR . Dengue, chikungunya, and Zika virus infections in Latin America and the Caribbean: a systematic review. Rev Panamericana Salud Publ = Pan Am J Public Health. (2023) 47:e34. doi: 10.26633/rpsp.2023.34. PMID: 36788963 PMC9910557

[75] Wen JS Jiang LF Zhou JM Yan HJ Fang DY . Computational prediction and identification of dengue virus-specific CD4(+) T-cell epitopes. Virus Res. (2008) 132:42–8. doi: 10.1016/j.virusres.2007.10.012. PMID: 18061300 PMC7114202

